# Comparative evaluation of performance and precision of multiplex immunoassays measuring human IgG to *Streptococcus pyogenes* vaccine antigens

**DOI:** 10.3389/fimmu.2026.1810546

**Published:** 2026-04-23

**Authors:** Michael Morici, Jill Gilmour, Omar Rossi, Martina Carducci, Zac Dempsey, Luisa Massai, Luca Rovetini, Matthew Cooper, Michael Serralha, Hannah Frost, Danilo Gomes Moriel, Jonathan Carapetis, Andrew Steer, Alma Fulurija

**Affiliations:** 1 The Australian Strep A Vaccine Initiative (ASAVI), Australia; 2 Wesfarmers Centre for Vaccines and Infectious Diseases, The Kids Research Institute Australia, Nedlands, WA, Australia; 3 Faculty of Medicine, Imperial College of Science Technology and Medicine, London, United Kingdom; 4 JWG Global Lab Solutions, Dumfries, United Kingdom; 5 GSK Vaccines Institute for Global Health (GVGH) S.r.l., Siena, Italy; 6 Centre for Child Health Research, University of Western Australia, Crawley, WA, Australia; 7 Tropical Diseases Research Group, Murdoch Children’s Research Institute, Melbourne, VIC, Australia; 8 Department of Paediatrics, Perth Children’s Hospital, Nedlands, WA, Australia; 9 Department of Paediatrics, University of Melbourne, Melbourne, VIC, Australia; 10 Department of Infectious Diseases, Royal Children’s Hospital Melbourne, Melbourne, VIC, Australia; 11 School of Biological and Medical Sciences, University of Western Australia, Crawley, WA, Australia

**Keywords:** analytical performance, antibody quantification, assay qualification, bead-based immunoassay, electrochemiluminescence, method comparison, multiplex immunoassay, *Streptococcus pyogenes* (GAS)

## Abstract

**Introduction:**

*Streptococcus pyogenes* (Group A Streptococcus, Strep A) is a Gram-positive bacterium and one of the most significant global bacterial pathogens, with disproportionate disease burden affecting low and middle-income countries. Despite substantial mortality and immune sequelae caused by Strep A, no licensed vaccines exist, and correlates of protection remain unestablished. Advancing Strep A vaccine development requires standardized and qualified assays to accurately measure vaccine-induced immune responses to support vaccine development and licensure/post-licensure monitoring. This study reports a direct comparison of two qualified multiplex immunoassays designed to detect human IgG antibodies targeting four distinct Strep A vaccine antigens.

**Methods:**

The MSD assay platform employs positional multiplexing with electrochemiluminescent detection, while the Luminex system uses bead-based multiplexing with fluorescent detection. Both assays used standardized intravenous immunoglobulin dilutions as reference standards. Assay precision was evaluated by analyzing eight samples from human volunteers selected to span the dynamic range for the four antigens Streptolysin O, *S. pyogenes* cell envelope protein, *S. pyogenes* adhesion and division protein, and group A carbohydrate.

**Results:**

Both platforms demonstrated comparable intermediate precision and repeatability profiles. Increased variance was observed in samples with very low antibody concentrations. Neither operator variability nor day-to-day factors significantly impacted assay results. Additionally, inter-assay coefficients of variation for standards remained below 15% within the pre-established quantitative ranges for both platforms.

**Discussion:**

The findings demonstrate that both multiplex immunoassays show equivalent precision and repeatability for quantifying human IgG to four Strep A vaccine antigens, making both platforms suitable for supporting early clinical trials and seroepidemiological studies.

## 1 Introduction

*Streptococcus pyogenes* (Group A Streptococcus, Strep A) is responsible for a wide spectrum of clinical manifestations, ranging from pharyngitis to severe invasive diseases, and remains a major cause of global morbidity and mortality (1). The substantial global disease burden attributable to Strep A infections underscores the urgent need for effective vaccine strategies. For many licensed vaccines, including meningococcal and pneumococcal formulations, the identification of biomarkers predictive of protection has accelerated clinical development and regulatory approval (2, 3). However, such correlates of protection are lacking for Strep A (4) and will likely not be established until vaccine candidates demonstrate protective efficacy in large-scale clinical trials. Consequently, the Strep A vaccine field requires standardized, qualified immunoassays to accurately assess and compare different vaccine-induced immune responses parameters, such as the responder rate and magnitude and duration of the response, more particularly for conserved vaccine antigens, including Streptolysin O (SLO), *S. pyogenes* cell envelope protein (SpyCEP), *S. pyogenes* adhesion and division protein (SpyAD), and group A carbohydrate (GAC). These standardized and qualified assays are essential for optimizing dosing regimens, adjuvant selection and identifying the most promising vaccine candidates to advance through safety and efficacy trials and licensure.

Several technologies exist to measure antigen-specific antibody responses, ranging from more traditional monoplex binding techniques such as ELISA (5) to various multiplex platforms using fluorescent (6) or electrochemiluminescent (7) detection systems. Each platform employs distinct reagents and detection methodologies, potentially yielding different performance characteristics related to assay precision, reproducibility, and sensitivity. Multiplexing platforms offer significant advantages over monoplex ELISAs, including enhanced dynamic range, higher throughput, and improved scalability, while being sample-sparing, which is pivotal for disease indications such as Group A Streptococcus where target populations for future vaccines will be very young children, in possibly hard-to-reach settings.

In preparations for Phase I trials of the GSK 4 component vaccines (registration number NCT07085702) two multiplex immunoassays were independently developed and fully qualified and fit for purpose, to quantify IgG responses to the vaccine antigens using Luminex and MSD multiplexing platforms (8–16), (Gilmour et al., in preparation). While assays on both platforms demonstrated excellent specificity, linearity, and precision with documented limits of detection and quantification in their respective validation studies, no direct comparison of their performance had been conducted. In this study we present a head-to-head comparison of the two multiplex platforms, evaluating IgG responses to the four conserved S*. pyogenes* antigens matched to vaccine— SLO, SpyCEP, SpyAD, and GAC—using the validated methods, identical reference standard materials and samples to rigorously assess and compare their performance. The aim of the study was to compare the validated assays with a focus on precision, and select one to assess vaccine trials. The aim was not to revalidate the assays. Assays, including curve construction and fitting, sample dilutions, and sample interpolations, were carried out according to the respective qualified methods.

## 2 Materials and methods

### 2.1 Electrochemiluminescence based immunoassay

The six-plex MSD assay was carried out as previously described (10, 12). Purified GAC and purified recombinant proteins SLO, SpyCEP, and SpyAD were produced and fully characterized by GSK Vaccines Institute for Global Health (GVGH), while recombinant ScpA and DNaseB were obtained from GenScript. Antigens were printed by MSD (USA) and bound directly to pre-specified carbon electrode spots on 10-spot 96-well plates (see **Supplementary Table 1** for coating concentrations). Samples were measured against a 12-point calibration curve constructed from serially diluted intravenous immunoglobulin (IVIG) (CreativeBiomart, cat. THP-0108), with standards (including standard 12, a blank which is also used to monitor background) run in duplicate wells and samples run in quadruplicate wells (see **Supplementary Figure 1** for plate layout.) The analysis compared the results from the four vaccine antigens only (SLO, SpyCEP, SpyAD, and GAC). The two additional antigens in the MSD multiplex, DNaseB and ScpA, have been shown not to affect measurement of the four vaccine antigens (Gilmour et al., in preparation).

All test samples were diluted to 1:100,000 in MSD Diluent 100, which was selected as the optimal dilution based on dilution linearity experiments as detailed in a manuscript currently under preparation (Gilmour et al., in preparation). MSD six-plex assay plates were blocked for 30 minutes with MSD Blocker A Solution, washed with MSD wash buffer using a Tecan HydroSpeed plate washer, and loaded with 50 µl/well of each sample, standard, and control. The plates were incubated for two hours, shaking at room temperature. Plates were then washed three times with 300 µl/well of wash buffer, and 50 µl of 1 µg/mL anti-human IgG SULFO-TAG detection antibody solution was added to each well. Plates were incubated for one hour under shaking at room temperature and then washed again with MSD wash buffer. MSD GOLD Read Buffer B was added (150 µl/well) and plates were immediately read with a MSD Sector S 600 instrument. Raw data processing was performed in MSD Discovery Workbench software (version 4). Standard curves were generated using four parameter logistic regression curve fitting with 1/y^2^ weighting, as established during development and of the assay qualification (Gilmour et al., in preparation). Quantitative MSD assay results are reported in arbitrary units per milliliter (AU/mL) relative to the standard curve. Analytical performance parameters including limits of quantitation and the limit of blank were previously established during the assay development and qualification (**Supplementary Table 2**). In instances where the limit of blank exceeded the lower limit of quantitation, the greater of these two values was designated as the effective lower quantitative limit of the assay.

### 2.2 Bead-based immunoassay

The four-plex bead-based immunoassay using Luminex technology was carried out as previously described (9). In brief, MagPlex magnetic beads were coupled to fully analytically characterized SLO, SpyCEP, SpyAD, and biotinylated GAC. All coupling procedures were performed by GVGH laboratory, and coupled beads were supplied to the ASAVI Central Immunogenicity Laboratory (CIL) at The Kids Research Institute Australia for the comparison study. The coupling chemistry employed carboiimide-mediated conjugation (NHS-EDC) to immobilize 10 µg of SLO and SpyCEP and 20 µg of SpyAD and streptavidin on to 1.25×10^6^ magnetic microspheres (bead regions 30, 20, 12, and 25, respectively). Biotinylated GAC was subsequently captured on the streptavidin-coupled beads through high-affinity biotin-streptavidin interaction. Calibration curves were constructed using ten three-fold serial dilutions of IVIG, and a blank was included on each plate. An initial verification run was conducted using a Privigen IVIG lot previously employed to generate standard curves for the Luminex assay in the GVGH laboratory, confirming assay operation within the expected parameters and enabling comparison with a calibration curve made from IVIG sourced from CreativeBiomart (cat. THP-0108) (**Supplementary Figure 2**). Following confirmation of similar performance of the two IVIG materials, subsequent Luminex assays were conducted with the CreativeBiomart IVIG to ensure standardization of reference materials for both Luminex and MSD assays throughout the comparative study. For each assay, 50 µl of standards, samples or controls were added to appropriate wells followed by 10 µl of a bead mix containing 2,500 beads per region. Test samples were diluted to 1:3,000 in phosphate-buffered saline (PBS), followed by two additional three-fold serial dilutions performed directly in the assay plate to generate final dilutions of 1:9,000 and 1:27,000. Each set of three wells (1:3000, 1:9000, 1:27,000) was run in quadruplicate to mirror the quadruplicate measurements in the MSD assay. Plates were washed with PBS using a Tecan Hydrospeed automated plate washer with magnetic plate carrier, detection was carried out using a polyclonal goat anti-human IgG-R-phycoerythrin (PE) antibody (Jackson ImmunoResearch, cat. 109-116-097) and fluorescence was quantified using a Bio-Plex 200 instrument. Five-parameter logistic regression, previously established for the assay, was applied to fit the standard curves, and sample concentrations were calculated using Bio-Plex Manager software (version 6.1). Analytical performance parameters including limits of standard curve accuracy and the lower limit of quantitation were established during the assay qualification as previously reported (see **Supplementary Table 3**) (9). Where sample results are referred to as within or outside limits of standard curve accuracy, the comparison was made using the calculated concentration prior to dilution correction. All Luminex results are expressed in relative Luminex units per milliliter (RLU/mL).

### 2.3 Sample selection and preparation

Sample material was obtained from the Murdoch Children’s Research Institute (courtesy of Dr. Joshua Osowicki) from the CHIVAS-M75 study (17). The study, collection of samples, and related immunology research and assay development was approved by the Alfred Hospital Human Research Ethics Committee (500/17) and The Royal Children’s Hospital Melbourne Human Research Ethics Committee (77900). Five samples with the most comprehensive coverage of signal ranges for SLO, SpyCEP, SpyAD and GAC were strategically selected from a panel of 14 representative plasma samples which had been previously tested during MSD assay development as part of assessment of inter-laboratory reproducibility for transfer of the assay method (Gilmour et al., in preparation). The sample selection process ensured that there were individual samples with low, mid-range, and high antibody levels for each antigen. The number of samples tested was limited to enable testing in quadruplicate on both platforms in order to support a robust assessment of the precision of the assays, including intra-assay variability. Additionally, three pooled reference samples representing low, medium and high antibody concentrations across antigens (LC, MC, and HC samples, respectively), determined as part of MSD qualification (Gilmour et al., in preparation), were added to each plate for the comparison study.

To eliminate potential variability from freeze-thaw cycles, individual samples were thawed once, aliquoted into single use aliquots, and stored at ‑80 °C until testing. Prior to use, sample aliquots were thawed on ice, vortexed to ensure homogeneity, and then centrifuged at 10,000 × g at 4 °C for five minutes to remove any particulate.

Individual plasma sample preparation protocols were optimized for each platform’s requirements: a three-step serial dilution was used to prepare 1:100,000 dilutions for MSD assay, and a two-step serial dilution was used to prepare the 1:3000 dilutions for the Luminex assay which were then further diluted in the Luminex assay plate as described above. Pooled LC, MC, and HC samples were stored as ready-to-use, pre-diluted aliquots, and were thawed, vortexed briefly, and loaded directly without further dilution.

### 2.4 Assay precision testing

A systematic experimental design was implemented to evaluate inter-operator and intra-operator variability across multiple days for each assay platform. Two trained operators each performed Luminex and MSD assay runs in the ASAVI CIL at The Kids Research Institute on separate days, but with the same operators and structured testing schedule. For each assay method, operators performed one independent assay each on Day 1. On Day 2, one operator performed two independent assays, and on Day 3 the second operator performed two independent assays. On Day 4, each operator again performed one independent assay. Days were not all consecutive (**Supplementary Figure 3**).

### 2.5 Statistical analysis

For the Luminex platform, final concentration values were determined by calculating the median concentration across the three dilution levels (i.e., 1:3,000, 1:9,000, 1:27,000). The median calculation was applied only when at least two or more valid concentration values were available (that is, where data exists and/or are within the limits of detection), otherwise the sample value was excluded from analysis.

For both platforms, comprehensive statistical evaluations were performed to compare coefficients of variation (CVs), rank order of samples, repeatability, intermediate precision, and dynamic range for the assays.

Inter- and intra-plate CVs were calculated for the samples across each platform by taking the ratio of the sample standard deviation to the sample mean (reported as a percentage) with analysis restricted for values falling within the established quantitative limits of each assay (**Supplementary Tables S2**, **S3**). Inter-plate CVs were calculated for each platform from each plate’s mean concentration. Intra-plate CVs were calculated for each platform by calculating the mean of the CVs within each plate.

To assess intermediate precision and repeatability, the log_10_ transformed AU/mL or RLU/mL of replicates were analyzed using a random-effects statistical model that incorporated both day and operator as random effects, allowing calculation of the intermediate precision, repeatability and variance attributed to each factor via residual maximum likelihood method using MiniTab software. Coefficients of variance for repeatability and intermediate precision were subsequently calculated from the Log-transformed variance components and reverted to original AU/mL or RLU/mL with the equation $CV=\sqrt{e^{sLn^{2}}-1}$, where *sLn* represents the standard deviation of log-transformed variance components multiplied by Ln(10).

Dynamic range for each assay was calculated as the ratio of the upper quantitative limit to the lower quantitative limit using the established working ranges for each platform (limits of standard curve accuracy for Luminex, limits of quantitation/limit of blank for MSD).

## 3 Results

This study provides a direct comparison of the performance of two qualified multiplex immunoassay platforms for quantifying IgG responses to four conserved Strep A antigens. We evaluated a bead-based assay utilizing Luminex xMap technology and an electrochemiluminescent assay on the MSD platform. Both assays were specifically optimized for the detection of antibodies against SLO, SpyCEP, SpyAD, and GAC antigens, and have been previously qualified to support Strep A vaccine development initiatives.

The comparison study was conducted in a single laboratory, with both assays performed by two independent operators using identical calibrator material and a well-characterized panel of samples. The comparative analysis focused on three critical performance parameters: dynamic range, precision, and inter-platform correlation. Following successful method transfer to the ASAVI CIL, both assays were run successfully, and we systematically analyzed standard curve performance and assessed measurement precision for both individual and pooled samples. The resulting data provide valuable insights into the performance characteristics and interchangeability of these two analytical platforms to support Strep A vaccine development.

### 3.1 Dynamic range and standard curve accuracy

**Figure 1:**
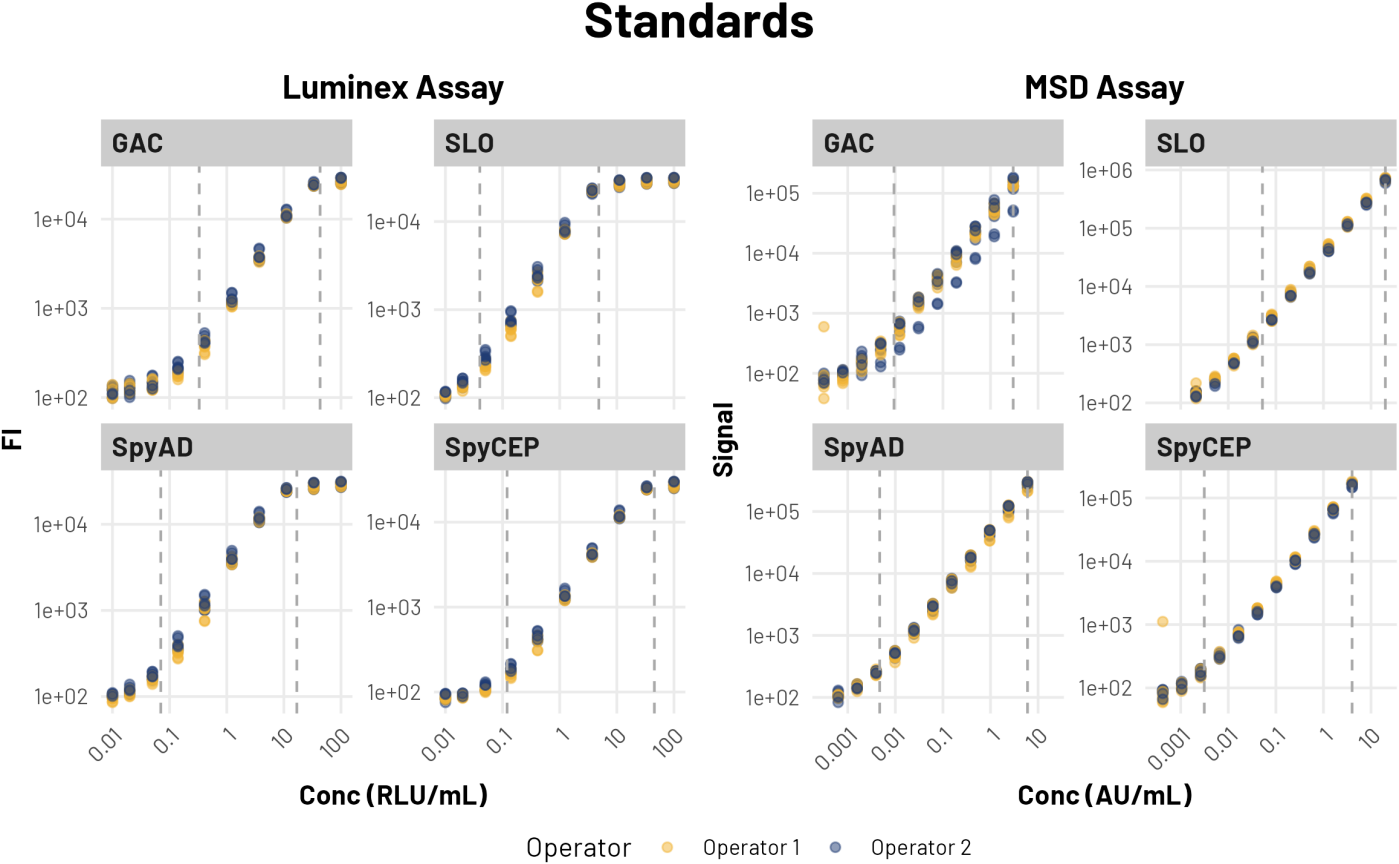
Standard curves from MSD and Luminex assays. Dashed lines indicate established upper and lower quantitative limits for the assay. Each dot represents a data point from a single well. Colors indicate operator. Plots show assigned concentration on the x-axis and signal on the y-axis.

The reference IVIG standard was employed as a calibrator for both multiplex assays, with each run including multiple dilutions in duplicate (**Table 1**). Both Luminex and MSD platforms demonstrated strong precision metrics, with all standard curve points showing acceptable inter- and intra-plate coefficients of variation (<20%) across all antigens (**Figure 1**).

**Table 1:** Comparison of standard curve parameters for Luminex and MSD assays.

Assay	Standard curve points	Standard curve dilution	IVIG concentration at top of curve (µg/mL)	Assigned units at top of curve
Luminex	10	3-fold	100.0	All assays: 100 RLU/mL
MSD	12^*^	2.5-fold	2.0	GAC: 3 AU/mL SLO: 20 AU/mL SpyAD: 6 AU/mL SpyCEP: 4 AU/mL

*The 12th point of the MSD curve is diluent only (0 concentration) and is included in the regression for curve fitting. Note that the Luminex assay includes a diluent only blank, which is used for blank subtraction and not counted in the standard curve points as it is not included in the regression.

The Luminex assay showed excellent inter-plate precision within its quantifiable range, with CVs below 10% for all protein antigens (range: 0.74-7.23%) and below 15% for GAC (range: 1.67-14.52%). The MSD platform exhibited even tighter precision parameters, with inter-plate CVs below 5% for all protein antigens (range: 0.84-4.45%) and below 10% for GAC (range: 2.53-6.55%).

Analysis of the dynamic ranges for all four antigens, calculated from established quantitative ranges for the assays, revealed that the MSD platform consistently provided broader quantitative capabilities across all antigens. Specifically, the MSD assay provided a dynamic range 0.39-0.73 Log units wider than that of the Luminex platform for the same antigens (**Table 2**), indicating enhanced capacity to resolve antibody concentrations across a wide range.

**Table 2:** Quantitative limits and dynamic ranges for MSD and Luminex assays.

Assay	MSD ULOQ (AU/mL)	MSD LLOQ/LOB (AU/mL)	Luminex upper LSCA (RLU/mL)	Luminex lower LSCA (RLU/mL)	MSD dynamic range (log)	Luminex dynamic range (log)
SLO	20	0.0522	4.84	0.04	2.583	2.083
SpyAD	6	0.00469	16.85	0.07	3.107	2.382
SpyCEP	4	0.00313	45.40	0.12	3.107	2.578
GAC	3	0.00938	43.05	0.33	2.505	2.115

Upper limit of quantitation (ULOQ) and lower limit of quantitation (LLOQ) or limit of blank (LOB), whichever was greater, are shown for the MSD assay. Upper limit of standard curve accuracy (LSCA) and lower LSCA are shown for Luminex. Dynamic range is shown as log_10_(upper limit/lower limit).

### 3.2 Precision assessment

**Figure 2:**
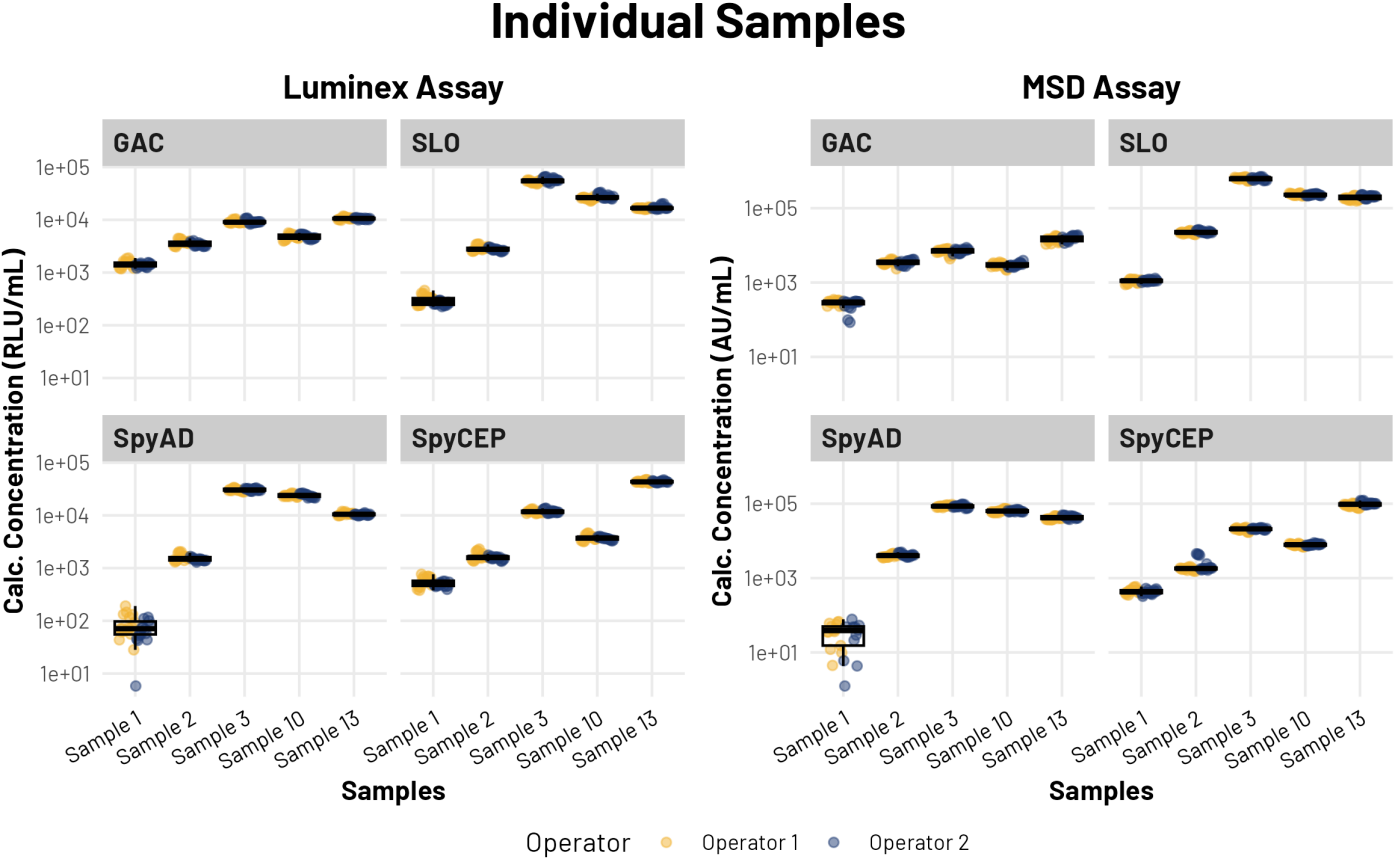
Individual sample results showing all data points which could be calculated, including data points below assay quantitative range for Sample 1 SpyAD. Colors indicate operator. Data points are arranged in chronological order for each operator’s runs. Box and whisker plots show median, first and third quartiles at the lower and upper hinges, and the smallest and largest values no further than 1.5 × the interquartile range from the respective hinges. Y-axes are log-scaled, in units for respective method (AU/mL for MSD, RLU/mL for Luminex.).

Five plasma samples with varying antibody levels were repeatedly tested, yielding inter- and intra-plate CVs below 15% for both Luminex and MSD assays. Sample 1, which consistently had the lowest antibody concentrations across the four antigens (**Figure 2**), predictably demonstrated the highest intra- and inter-assay variability (CVs). Nevertheless, both platforms demonstrated consistent sample measurements across several runs and between different operators (**Figure 2**).

The rank order of samples remained consistent between platforms for all antigens, with a single exception observed with sample 2 and sample 10 for the GAC antibody measurement. These two samples had similar GAC antibody concentrations and were ranked 2 and 3 compared with 3 and 2 (in ascending order from 1-5) (**Supplementary Table 4**) for the Luminex and MSD platforms, respectively.

Both repeatability and intermediate precision parameters remained below 20% CV for almost all samples across both assays (**Table 3**) confirming comparable and acceptable performance characteristics of the two assays. As expected, the sample with very low titers (Sample 1 for SpyAD) had high variability.

**Table 3:** Repeatability and Intermediate precision (IP) of four antigens for five individual samples and three pooled high, medium, and low-level samples in both MSD and Luminex assays. Values below LoQ or LLSCA were excluded.

Sample	Precision Component	SLO		GAC		SpyCEP		SpyAD	
		MSD	Luminex	MSD	Luminex	MSD	Luminex	MSD	Luminex
Sample 1	Repeatability	6.5	8.1	20.1	6.7	8.7	10.6	*126.6*	*56.1*
	IP	9.8	23.4	33.0	12.3	15.0	19.8	*146.9*	*71.5*
Sample 2	Repeatability	3.5	2.5	9.5	3.2	7.3	4.7	3.1	4.3
	IP	8.6	10.1	14.8	11.7	34.0	14.0	10.0	13.1
Sample 3	Repeatability	4.3	3.0	10.0	2.0	3.8	3.0	3.7	2.2
	IP	9.2	9.7	15.9	7.5	8.3	7.2	7.2	5.5
Sample 10	Repeatability	2.3	4.3	8.6	3.7	2.2	2.7	2.7	3.3
	IP	5.7	10.7	13.2	10.2	6.6	9.9	7.7	6.6
Sample 13	Repeatability	3.2	2.2	10.5	2.6	2.2	2.3	3.0	2.3
	IP	13.6	6.4	17.7	4.2	14.8	4.3	10.3	5.1
HC	Repeatability	6.6	2.5	6.7	4.0	5.6	2.5	7.2	3.7
	IP	8.1	8.6	10.5	7.7	8.0	10.4	9.4	9.3
MC	Repeatability	5.7	3.8	4.6	8.9	3.1	8.9	4.2	4.3
	IP	8.6	17.2	13.1	19.2	8.1	18.6	7.0	16.7
LC	Repeatability	5.1	5.7	7.7	23.5	3.2	28.4	5.6	8.3

For SLO, mean repeatability CVs were almost identical between platforms (3.96% for MSD and 4.02% for Luminex), and intermediate precision CVs were slightly lower for MSD (9.38%) than for Luminex (12.06%). SpyCEP measurement showed similar results with mean repeatability CVs of 4.84% and 4.66% and mean intermediate precision CVs of 15.74% and 11.04% for MSD and Luminex, respectively. SpyAD showed elevated variability for both repeatability and intermediate precision, primarily due to Sample 1. When Sample 1 was excluded, SpyAD demonstrated better precision metrics, with the lowest mean CVs for repeatability and intermediate precision for both platforms (both < 3.5% reproducibility and < 9% intermediate precision). GAC measurements showed slightly greater variability on the MSD platform compared with Luminex (mean repeatability 11.74% compared with 3.64% and mean intermediate precision 18.92% compared with 9.18% for MSD and Luminex, respectively).

Statistical analysis confirmed that neither operator variability nor run-to-run differences significantly influenced the assay outcomes for either platform (p-values > 0.05).

**Figure 3:**
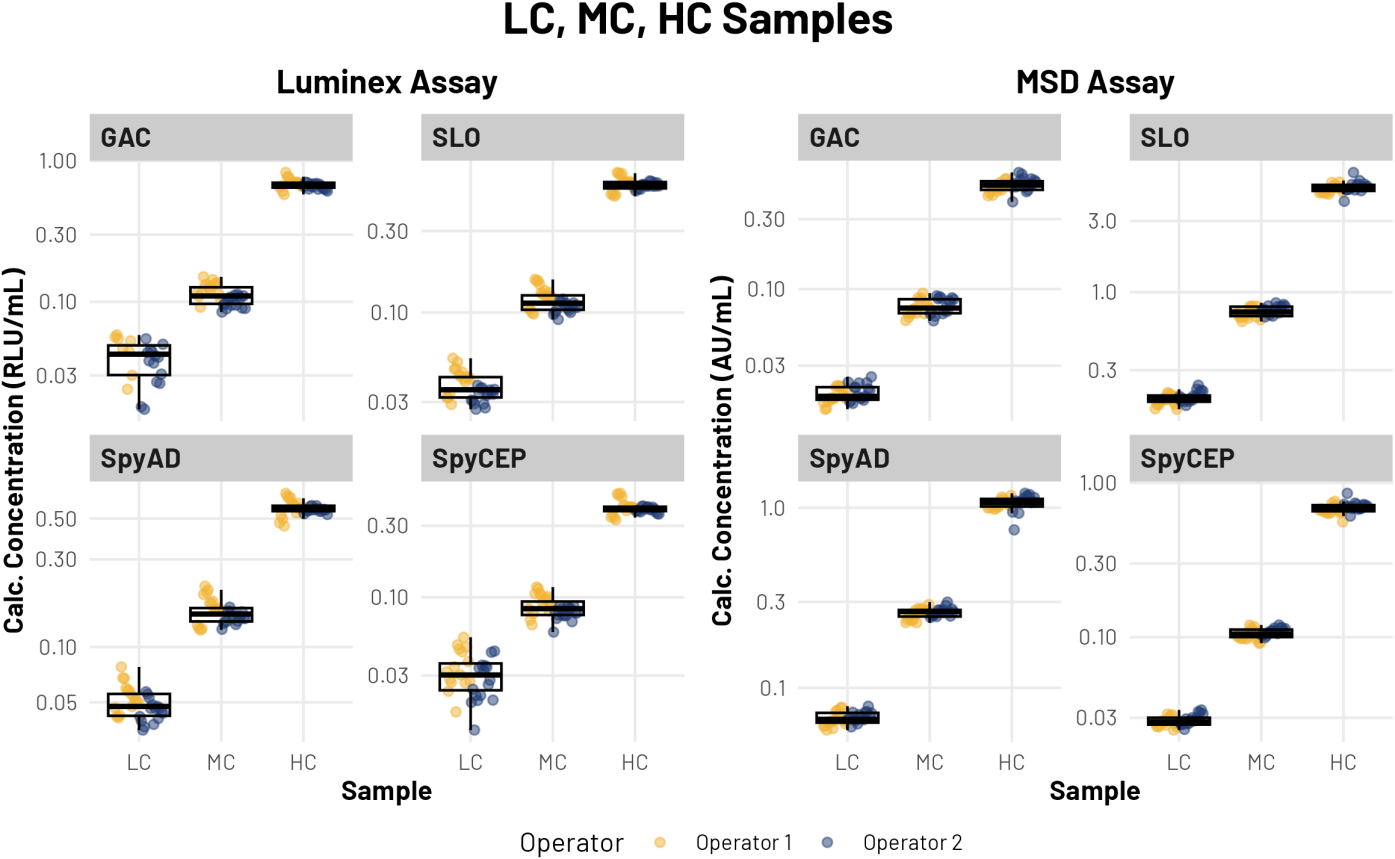
Pooled sample results in MSD and Luminex assays. Each plot shows calculated IgG concentrations for high (HC), medium (MC), and low (LC) pooled samples. Each point represents a calculated concentration from a single well. Each plate run had four replicate wells. Colors indicate operator. Data points are arranged in chronological order for each operator’s runs. Box and whisker plots show median, first and third quartiles at the lower and upper hinges, and the smallest and largest values no further than 1.5 × the interquartile range from the respective hinges.

Three additional pooled samples with low, medium and high antibody concentrations (referred to as LC, MC and HC samples, respectively) were included in the evaluation (**Figure 3**). These samples, originally selected as quality controls for the qualified MSD assay (as described in Gilmour et al., in preparation), fell within the quantitative limits for the MSD assay.

Inter- and intra-plate CVs on the MSD platform were below 10% for the protein antigens SLO, SpyCEP, and SpyAD (range 1.0-8.0%) and were below 15% for GAC (range 2.2-12.3%). On the Luminex platform, inter- and intra-plate CVs were below 20% for all repeated measurements that fell within the pre-established quantitative range (within the limits of standard curve accuracy (LSCA) prior to dilution correction). However, the MC sample fell below the Luminex assay lower LSCA (LLSCA) for GAC and SpyCEP, and the LC sample fell below the LLSCA for GAC, SpyCEP, and SpyAD, in line with the narrower dynamic range and requirement for more concentrated sample dilutions in the Luminex assay compared with the MSD assay. Repeatability CVs remained below 10% for the pooled HC and MC samples for all antigens across both Luminex and MSD platforms, with elevated values observed only for the LC sample in the Luminex assay for SpyCEP and SpyAD (**Table 3**). Intermediate precision CVs were below 11% for the HC sample across all antigens in both assays, and for the MC sample protein antigens in the MSD assay. For the MC sample in the Luminex assay, intermediate precision was reduced, consistent with the pre-diluted pooled sample falling near the lower end of the assay’s dynamic range.

## 4 Discussion

This study reports a robust comparison of performance of the Luminex and MSD multiplex immunoassays for quantifying IgG responses to four Strep A vaccine antigens with particular focus on assay precision. Given that both platforms have previously demonstrated accuracy, linearity and specificity, and have documented assay ranges and limits, we focused specifically on precision metrics. Both assays were independently developed using identical capture materials and were systematically compared using standardized reference materials and clinical samples by two operators working over multiple days in a single laboratory.

Prior studies have compared MSD and Luminex assays with traditional ELISAs (7, 18, 19), cytometric bead arrays (20) and against each other for both multiplexed cytokine measurements and human SARS-CoV-2 serology using commercially available assay kits (21–26). While these studies offer valuable insights to inform assay selection for research applications, the variability in reported cytokine assay ranges, sensitivity, and precision may reflect differences in proprietary or undisclosed antibodies and diluents used in the kits, rather than fundamental differences in the underlying multiplexing and detection technology. Despite the increasing adoption of multiplex immunoassays in vaccine development, limited comparative data exists specifically for MSD and Luminex platforms measuring specific human IgG antibodies. The work presented here addresses this knowledge gap by providing a direct comparison of these two technologies using identical antigens, clinical samples, and reference standards.

The use of a single source and lot of IVIG to construct standard calibration curves for both the Luminex and MSD assays enabled a direct comparison of the assay performance. As expected, the standards exhibited low intra- and inter-assay variability for both assays within their established quantitative ranges. This head-to-head comparison, using identical calibration standards and samples, enhances data comparability in the absence of an international reference standard. Notably, CV values for the standard curves were within acceptable limits for all antigens, with consistently lower variability observed for the protein antigens compared to the polysaccharide antigen (GAC) on both platforms.

Some differences were observed between the Luminex and MSD assays for the pooled MC and LC samples, with MC falling below the LLSCA for two of four antigens and LC falling below the LLSCA for three of four antigens in the Luminex assay. This outcome was consistent with expectations, as the pooled samples were originally developed and pre-diluted specifically for use in the MSD assay, which uses higher sample dilution factors and offers a larger dynamic range compared to the Luminex assay. Assay precision declining near the lower limit of quantification due to reduced signal-to-noise ratios is a well-established phenomenon in many assays, including immunoassays. This limitation can be mitigated by adjusting sample dilutions according to predefined acceptance criteria.

Both assay platforms demonstrated comparable consistency across individual samples. While we observed an unusually elevated SpyCEP value for Sample 2 in a single MSD assay run by Operator 2, other antibody measurements from the same well were unaffected. The data from this sample was retained in the analysis as no specific handling error could be identified, and the intra-run CV for the SpyCEP measurement remained acceptably low at 4.1%. Although sample CVs were mostly below 15%, they were generally slightly higher than CVs observed for the pooled HC, MC, and LC samples. Since pooled samples were stored pre-diluted, while individual samples required serial dilution from neat plasma for each plate, the difference suggests that the sample preparation process can influence assay precision and repeatability, although performance metrics for both assays remain within acceptable limits.

The rank order of sample antibody levels agreed was consistent between assay platforms for all samples and antigens, with the exception of a swap in position between samples 2 and 10 for the GAC measurement. Several factors could explain this discrepancy and may have influenced this result, including differences in detection antibodies, incubation periods, and washing conditions between the two assays. However, it remains unclear whether these variations in assay conditions, which could differentially impact lower-affinity antibodies, fully explain the observation. Notably, the traditional view that polysaccharide-directed antibodies are predominantly lower-affinity and restricted to the IgG2 subclass has been challenged recently (reviewed in (27)).

Additional considerations for selecting immunoassays platforms for vaccine immunogenicity studies include reagent stability, batch consistency, sample throughput, and cost per sample, though a rigorous evaluation of these factors was beyond the scope of the current study. A cost-per-sample comparison is inherently context-dependent, varying with the number of antigens, samples, geographic location, local labor costs, and available infrastructure (e.g., liquid handling systems).

This study provides insight into the comparative performance of Luminex and MSD assays for four Strep A vaccine antigens, however several limitations should be acknowledged when interpreting the results. First, while we selected samples to span the analytical range of both assays, a larger sample cohort would have allowed more robust comparison of the precision of the assays. Additionally, inter-laboratory reproducibility is an important consideration for multi-center clinical trials but was beyond the scope of the study. Notably, both the MSD and Luminex assays were successfully transferred to the ASAVI CIL hosted at The Kids Research Institute, Australia. For the MSD assay, a formal comparison of results generated at the MSD Assay Services laboratory (USA) and the ASAVI CIL was done. Similarly, the Luminex assay was verified at the ASAVI CIL using standardized IVIG reference materials with comprehensive data review conducted in collaboration with the GVGH laboratory. The successful inter-laboratory transfer and verification of both assay platforms further substantiates their robustness and reliability for vaccine development applications.

Both the MSD and Luminex assays can be readily adapted to measure specific IgG subclasses and alternative immunoglobulin isotypes through the use of subclass and isotype-specific detection antibodies. Given the growing relevance of IgG subclass analysis in vaccine development and epidemiological research, our results suggest both technologies provide suitable and flexible platforms for these expanded applications.

When selecting assay platforms, consideration should be given to factors including production batch size, sample throughput, cost efficiency, dynamic range requirements, reagent stability and shelf-life, and scalability to support the entire clinical development pathway. Each platform offers distinct advantages and limitations that may be relevant depending on the clinical phase and testing laboratory capabilities. **Supplementary Figure 4** provides proposed plate layouts for vaccine trial sample testing for both platforms, offering practical guidance for implementation.

In conclusion, both Luminex and MSD multiplex immunoassays demonstrated comparable robustness, precision, and performance characteristics for the four Strep A vaccine antigens examined in this study, confirming the suitability of either assay platform for immunogenicity assessments during vaccine trials.

## Data Availability

The raw data supporting the conclusions of this article will be made available by the authors, without undue reservation.
