# Supplementary material for: Comparative evaluation of performance and precision of multiplex immunoassays measuring human IgG to Streptococcus pyogenes vaccine antigens
Source: Front Immunol. 2026 Apr 23;17:1810546. doi: 10.3389/fimmu.2026.1810546 (PMC13149258; doi:10.3389/fimmu.2026.1810546)
Supplement: Supplementary file 1 [file DataSheet1.docx]

Supplementary Material

# Supplementary Data

**Figure S1:** Plate layouts for Luminex and MSD assays used in the study


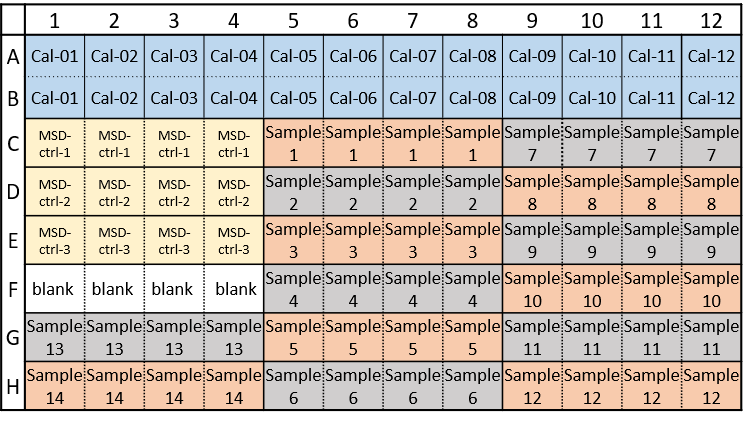


MSD

Luminex


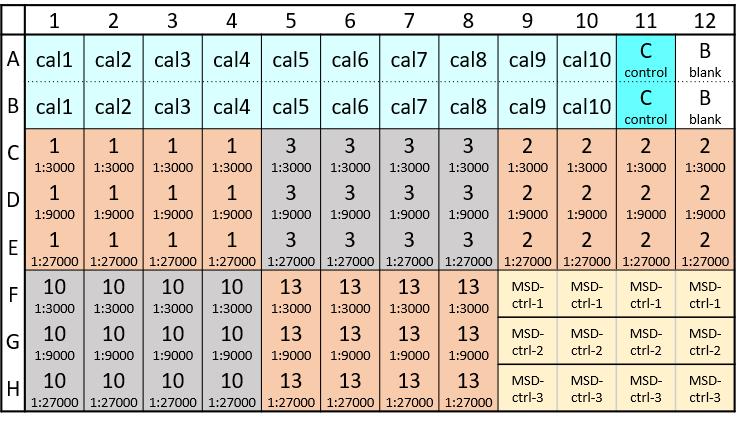


Quadruplicate measurement

Median calculated value from each

| **Spot #** | **Capture Reagent** | **Antigen Source** | **Coating Conc. (μg/mL)** |
| --- | --- | --- | --- |
| **1** | **GAC** | **GVGH** | **50** |
| **2** | **DNase B** | **GenScript** | **100** |
| **3** | **SLO** | **GVGH** | **50** |
| **8** | **SpyAD** | **GVGH** | **100** |
| **9** | **ScpA** | **GenScript** | **50** |
| **10** | **SpyCEP** | **GVGH** | **100** |

Table S1: Antigen source and coating concentrations for MSD assay plates

| **Assay** | **ULOD/ULOQ (AU/mL)** | **LLOQ (AU/mL)** | **LOB (AU/mL)** | **Lower Quantitative Limit (AU/mL)** |
| --- | --- | --- | --- | --- |
| GAC | 3 | 0.00938 | 0.0038 | 0.00938 |
| SLO | 20 | 0.01563 | 0.0522 | 0.0522 |
| SpyAD | 6 | 0.00469 | 0.00117 | 0.00469 |
| SpyCEP | 4 | 0.00313 | 0.00209 | 0.00313 |

**Table S2:** Established limits of quantitation for MSD assay.

| **Assay** | **ULSCA (RLU/mL)** | **LLSCA (RLU/mL)** | **LLOQ**  **(RLU/mL)** |
| --- | --- | --- | --- |
| GAC | 43.05 | 0.33 | 99 |
| SLO | 4.84 | 0.04 | 12 |
| SpyAD | 16.85 | 0.07 | 21 |
| SpyCEP | 45.4 | 0.12 | 36 |

**Table S3**: Established upper and lower limits of standard curve accuracy (ULSCA and LLSCA, respectively) and lower limit of quantitation (LLOQ) for the Luminex assay [1].

**Figure S2:** Luminex standard curves: comparison of Privigen and MSD (CreativeBiomart) IVIG

**Figure S3:** Assay comparison run plans

Run separately for each platform:

**Table S4:** Test Sample Rank Order

| **Assay** | **Platform** | **sample 1** | **sample 2** | **sample 3** | **sample 10** | **sample 13** |
| --- | --- | --- | --- | --- | --- | --- |
| SLO | Luminex | 1 | 2 | 5 | 4 | 3 |
|  | MSD | 1 | 2 | 5 | 4 | 3 |
| GAC | Luminex | 1 | 2 | 4 | 3 | 5 |
|  | MSD | 1 | 3 | 4 | 2 | 5 |
| SpyAD | Luminex | 1* | 2 | 5 | 4 | 3 |
|  | MSD | 1* | 2 | 5 | 4 | 3 |
| SpyCEP | Luminex | 1 | 2 | 4 | 3 | 5 |
|  | MSD | 1 | 2 | 4 | 3 | 5 |

Rank order of individual test samples (ascending: 1 = lowest titre, 5 = highest titre)

*SpyAD sample 1 was below assay quantitative limits

**
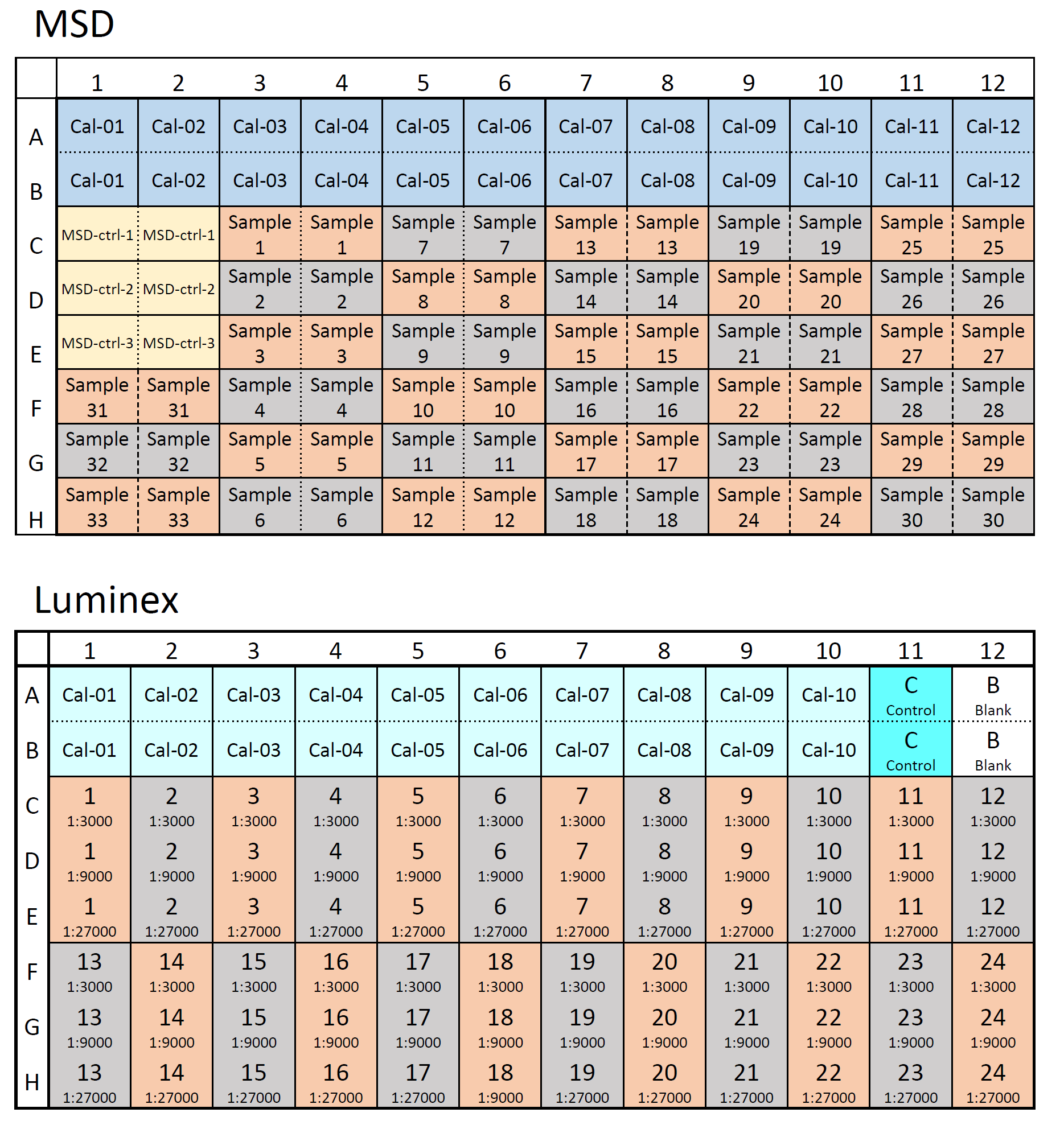
Figure S4:** Proposed plate layouts for testing samples in clinical trials

References

1. Keeley, A.J., et al., *Development and Characterisation of a Four-Plex Assay to Measure Streptococcus pyogenes Antigen-Specific IgG in Human Sera.* Methods and Protocols, 2022. **5**(4): p. 55.
